# Radiation dose reduction during adrenal vein sampling using a new angiographic imaging technology

**DOI:** 10.1038/s41598-022-09984-2

**Published:** 2022-04-12

**Authors:** Clemens Spink, Maxim Avanesov, Alexander Lenz, Frank Oliver Henes, Lennart Well, Thomas Schmidt, Gerhard Adam, Harald Ittrich, Peter Bannas

**Affiliations:** 1 Department of Diagnostic and Interventional Radiology and Nuclear Medicine, University Medical Center Hamburg-Eppendorf, Hamburg, Germany; 2 Philips Healthcare, Hamburg, Germany

**Keywords:** Endocrine system and metabolic diseases, Medical imaging, X-ray tomography, Endocrine system, Adrenal glands

## Abstract

To compare the patient radiation doses during angiographic selective adrenal vein sampling (AVS) before and after an imaging technology upgrade. In this retrospective single-center-study, cumulative air kerma (AK), cumulative dose area product (DAP), fluoroscopy time and contrast agent dosage were recorded from 70 patients during AVS. 35 procedures were performed before and 35 after an imaging processing technology upgrade. Mean values were calculated and compared using an unpaired student’s t-test. DSA image quality was assessed independently by two blinded readers using a four-point Likert scale (1 = poor; 4 = excellent) and compared using Wilcoxon signed-rank test. After the technology upgrade we observed a significant reduction of 35% in AK (1.7 ± 0.7 vs. 1.1 ± 0.7 Gy, p = 0.01) and a significant reduction of 28% in DAP (235.1 ± 113 vs. 170.1 ± 94 Gy*cm^2^, p = 0.01) in comparison to procedures before the upgrade. There were no significant differences between the number of exposure frames (143 ± 86 vs. 132 ± 61 frames, p = 0.53), fluoroscopy time (42 ± 23 vs. 36 ± 18 min, p = 0.22), or the amount of contrast medium used (179.5 ± 84 vs. 198.1 ± 109 ml, p = 0.41). There was also no significant difference regarding image quality (3 (2–4) vs. 3 (2–4), p = 0.67). The angiographic imaging technology upgrade significantly decreases the radiation dose during adrenal vein sampling without increasing time of fluoroscopy or contrast volume and without compromising image quality.

## Introduction

Primary aldosteronism (PA) is the most frequent secondary form of hypertension and is characterized by an autonomous adrenocortical over secretion of aldosterone^1^. PA has a prevalence of up to 11% in hypertensive patient populations^2, 3^. The aldosterone excess either results from bilateral idiopathic adrenal hyperplasia (IHA) or unilateral aldosterone-producing adenoma (APA)^4^.

The appreciation that primary aldosteronism is far more common than previously recognized and the essential distinction between APA and IHA results in an increasing requirement for adrenal vein sampling^5^.

Angiographic selective adrenal vein sampling (AVS) allows identification of patients with primary aldosteronism by direct sampling of hormones produced by each gland^8^. Patients with APA can benefit from adrenalectomy, while IHA-patients require conservative drug treatment^4^. Removing the identified gland producing excess adrenal hormone may cure secondary hypertension, thereby significantly improving patient’s prognosis regarding cardiovascular complications and renal function^9^.

Unfortunately, diagnostic angiographic procedure time is relatively long because AVS is a technically demanding procedure: AVS may be prolonged because of anatomic variations of adrenal vein ostia, particularly of the right adrenal vein^10^. A prolonged search for venous ostia can result in long fluoroscopy times up to 184 min with high dose area products (DAP) up to 3181 Gy*cm^2^^11^.

Different techniques of radiation dose reduction for angiographic procedures have been developed over the past years^12–15^. Particularly the potential of noise reduction by image-processing have been successfully introduced and recently advanced further^16^. Real-time post-processing imaging algorithms allow reduced radiation doses, while maintaining adequate image quality^17^. However, the degree of dose reduction varies and depends on the examined body region. Specific post-processing imaging algorithms offer tailor-made settings depending on the particular field of intervention, such as noise reduction, motion compensation and improved edge imaging. Motion compensation is extremely useful during thoracic or upper abdominal interventions in order to reduce breathing artifacts. Algorithms for stable interventions such as cerebral or peripheral angiography mainly benefit from noise reduction and improved edge imaging. Published dose reductions range from 43% during cardiac interventions up to 83% during iliac angiography^16–18^. Furthermore, significantly reduced radiation doses up to 59% during bronchial artery embolization as well as up to 57% during intrahepatic interventions such as transjugular intrahepatic portosystemic shunts have been reported^19–21^.

Up to now, the dose reduction potential of the AlluraClarity upgrade has not been assessed in patients undergoing AVS. The aim of our study was therefore to compare radiation doses in patients undergoing AVS between AlluraClarity and the precursor technology.

## Materials and methods

### Study design and patient selection

Our retrospective single-center-study was approved by the local institutional review board “Aerztekammer Hamburg”. Informed consent of all study participants had been obtained. All procedures were performed in accordance with the standards of the act for healing professions of Hamburg, Germany with the principles of the 1964 Declaration of Helsinki and its following amendments. All clinical imaging data were anonymized. The anonymization of patient data in the research process ensured data protection in accordance with the European General Data Protection Regulation.

Between June 2012 and August 2018, a total of 91 AVS procedures have been performed at our department on patients with suspected primary aldosteronism using an Allura FD20 angiographic system (Philips Healthcare, Best, Netherlands).

In June 2014 the angiographic image-processing technology received an upgrade from “Allura Xper” to “AlluraClarity” (Philips Healthcare, Best, The Netherlands).

Our *study group* was recruited from 44 patients undergoing AVS between June 2014 and August 2018 after the AlluraClarity upgrade. Nine of these 44 patients (20.5%) had to be excluded due to incomplete unilateral left adrenal vein sampling. The remaining 35 patients with successful sampling of both left and right AVS during a single AVS procedure resulted in our *study group*.

Our *control group* was recruited from 47 patients undergoing AVS between June 2012 and May 2013 before the AlluraClarity upgrade. Twelve of these 47 patients (25.5%) had to be excluded due to incomplete unilateral left adrenal vein sampling. The remaining 35 patients with successful sampling of both left and right adrenal veins during a single AVS procedure resulted in our *control group* (Table 1).

**Table 1:** Patient demographics.

Patient characteristics	Allura Xper	AlluraClarity	p-value
Number of patients	35	35	
Female	15 (42%)	16 (45%)	
Age (y)	58.1 ± 13.2	54.4 ± 9.5	0.13
Weight (kg)	76.4 ± 12.5	77.1 ± 13.1	0.74
Size (m)	1.6 ± 0.2	1.6 ± 0.1	1.00
Body mass index (kg/m^2^)	27.1 ± 5.2	28.8 ± 3.9	0.35

Data presented as mean ± standard deviation; * = significant.

None of the included patients underwent AVS twice.

### Adrenal vein sampling (AVS)

AVS is a standardized procedure at our institution in accordance to the Endocrine Society guidelines and expert consensus statement of the American Heart Association^6, 7^. Aldosterone release was stimulated before and during AVS with synacthen, a synthetic signal peptide of adrenocorticotropic hormone (ACTH) in a 50 μg/h infusion. The ACTH infusion prior the AVS provides the advantage of constant adrenal stimulation which levels circadian deviation^6^.

Sampling was performed in all cases by board certified radiologists with 8–20 years of experience in interventional radiology. Details of the procedure are described in detail elsewhere^5^. First, blood samples (5 ml) from the inferior vena cava (IVC) were collected as a reference. Second, the left adrenal vein ostium was carefully probed selectively with 5F-Cobra or 5F-Aachen-I catheters (Radifocus, Terumo, Japan). Third, the right adrenal vein ostium was carefully probed selectively with a 5F-Mikaelsson catheter (Impress, Merit Medical, USA) or 5F/4F-Sidewinder or 5F-Cobra catheters (Radifocus, Terumo, Japan). Venograms were performed on both left and right adrenal veins using 3–5 ml contrast agent (Imeron 300, Bracco, Italy) to document successful probing of adrenal vein ostia. Blood samples (5 ml) were collected from each adrenal vein ostium. Intraprocedural cortisol measurements confirmed successful adrenal vein sampling. Finally, IVC blood samples (5 ml) were collected as a second and final reference.

The amount of contrast agent was recorded for each procedure. Medical notes records were reviewed to collect information on patient’s demographics, such as age as well as weight and size for calculation of body mass index (BMI) (Table 1).

### Imaging systems

The imaging technology AlluraClarity upgrade improves noise reduction by both optimized hardware and real-time image processing-algorithms, tailor-made for different body areas by adjusted acquisition parameters^16^. Both systems, Allura Xper and AlluraClarity, have a 1 mm aluminum filter combined with a copper filter that can be 0.1, 0.4, or 0.9 mm thick. Compared to the precursor technology AlluraClarity provides a new default setting of additional filtration according to the different fluoroscopy levels (low, medium or high) (Table 2). Further, during fluoroscopy the nominal tube voltage is decreased from 80 to 70 kV. Different fluoroscopy settings ranged from low to medium to high dose include increasing frames per second (fps) and decreasing levels of filtration. After the technology upgrade, the pulse width was halved from 7.0 to 3.5 ms resulting in shortened pulses during fluoroscopy. The focal spot size was decreased from 7.0 to 3.5 mm during DSA to improve spatial resolution.

**Table 2:** Imaging settings for abdominal fluoroscopy and DSA.

	Allura Xper						AlluraClarity					
	Frames per second (fps)	Inherent filtration (mm)	Additional filtration (mm)		Limitation patient entrance doserate [mGy/s]	Detector doserate [nGy/s]	Frames per second (fps)	Inherent filtration (mm)	Additional filtration (mm)		Limitation patient entrance doserate [mGy/s]	Detector doserate [nGy/s]
		Al	Cu	Al				Al	Cu	Al		
**Fluoroscopy**												
low dose	7.5	3	0.9	1	349	250	15	3	0.9	1	175	200
medium dose	15	3	0.4	1	697	250	15	3	0.4	1	435	250
high dose	15	3	0.1	1	1395	250	15	3	0.1	1	1020	250
Pulse width (ms)	7						3.5					
Tube current (mA)	160						60					
Tube voltage (kV)	80						70					
**DSA**												
	fps	Al	Cu	Al	Detector Dose per frame [nGy/fr]		fps	Al	Cu	Al	Detector Dose per frame [nGy/fr]	
Default	3	3	0.1	1	2150		2	3	0.1	1	1000	
Focal spot (mm)	7						3.5					
Tube current (mA)	160						60					
Tube voltage (kV)	80						80					

Apart from hardware changes, the new technology uses different background post-processing algorithms during image acquisition. These algorithms include temporal and spatial noise reduction techniques as well as motion compensation and improved edge imaging^17^.

### Radiation dose measurement and documentation

The dose area product (DAP) defines the amount of dose absorption of an irradiated area in Gy*cm^2^, measured by ionization chambers placed nearby X-ray collimators^23^. It is important to note, that the DAP referring to the air kerma-area product can be taken as a dosimetric indicator for patient exposition. The DAP is independent of the distance from the radiation source.

Kerma, the kinetic energy released in matter can be calculated in the air at the interventional reference point (IRP) in units of Gray (Gy) depending on the image system acquisition parameters of the field collimation, the tube voltage, the tube current, and the source to image-receptor distance. The IRP is located along the X-ray beam at a distance of 15 cm above the isocenter in direction from the X-ray tube towards the detector. Therefore, the air kerma (AK) can be used as a dosimetric indicator for estimation of the patient’s skin dose^22^.

During each interventional procedure different parameters including AK, DAP, the number of exposure frames, and the fluoroscopy time were collected automatically within Radiation Dose Structured Reports (RDSR) (Table 3). After each intervention these reports were transferred into the Picture Archiving and Communication System (PACS)^24^.

**Table 3:** Procedure parameters during AVS.

Parameters	Allura Xper	AlluraClarity	Percentage difference	p-value
Cumulative DAP (Gycm^2^)	235.1 ± 113.1	170.1 ± 94.5	−28%	0.01*
Cumulative AK (Gy)	1.7 ± 0.7	1.1 ± 0.7	−35%	0.01*
Number of exposure frames	143 ± 86	132 ± 61	−8%	0.53
Fluoroscopy time (min)	42.3 ± 23.1	36.5 ± 18.6	−14%	0.22
Contrast agent volume (ml)	179.5 ± 84.0	198.1 ± 109.1	+ 10%	0.41

Data presented as mean ± standard deviation, * = significant.

### Image quality assessment

Qualitative DSA image analysis was performed by two radiologists with 3 years of experience in interventional radiology each. Both readers were blinded to the imaging technology and assessed randomized venograms of adrenal veins. Both readers used the same working station monitor with maintained window and image settings at default alignments. The two readers determined separately whether each venogram satisfied the following findings: sharp delineation of the adrenal veins with sufficient contrast, contrast stain of the adrenal gland, low level of subjective image noise. The readers scored the images using a four-point Likert scale adapted from Morita et al.^13^: 4 = excellent, all findings were observed; 3 = good, two of the findings were observed; 2 = fair, one of the findings was observed; 1 = poor, no findings were observed.

### Statistical analysis

Mean or median values of recorded values as well as standard deviations (SD) or ranges were calculated as appropriate. Results of AK, DAP, fluoroscopy time, age and BMI were compared using an unpaired student’s t-test. Results of Likert scale variables were compared using a Wilcoxon-Mann–Whitney test. Cohen’s weighted kappa was calculated for inter-rater reliability. P-values < 0.05 were considered statistically significant. Statistical analyses were performed with Quick Calcs 2015 (Graphpad Software, La Jolla, CA) and SPSS 22.0 (IBM Corp., Armonk, NY).

## Results

### AVS procedures

There was no significant difference regarding age (54.4 ± 9.5 vs. 58.1 ± 13.2 y, p = 0.13) or BMI (28.8 ± 3.9 vs. 27.1 ± 5.2 kg/m^2^, p = 0.35) between the study group (AlluraClarity) and the *control group* (Allura Xper) (Table 1). There was no significant difference regarding the amount of contrast medium used (198 ± 109 vs. 179 ± 84 ml, p = 0.41) between the study group (AlluraClarity) and the *control group* (Allura Xper) (Table 3).

### Radiation dose

**Figure 1:**
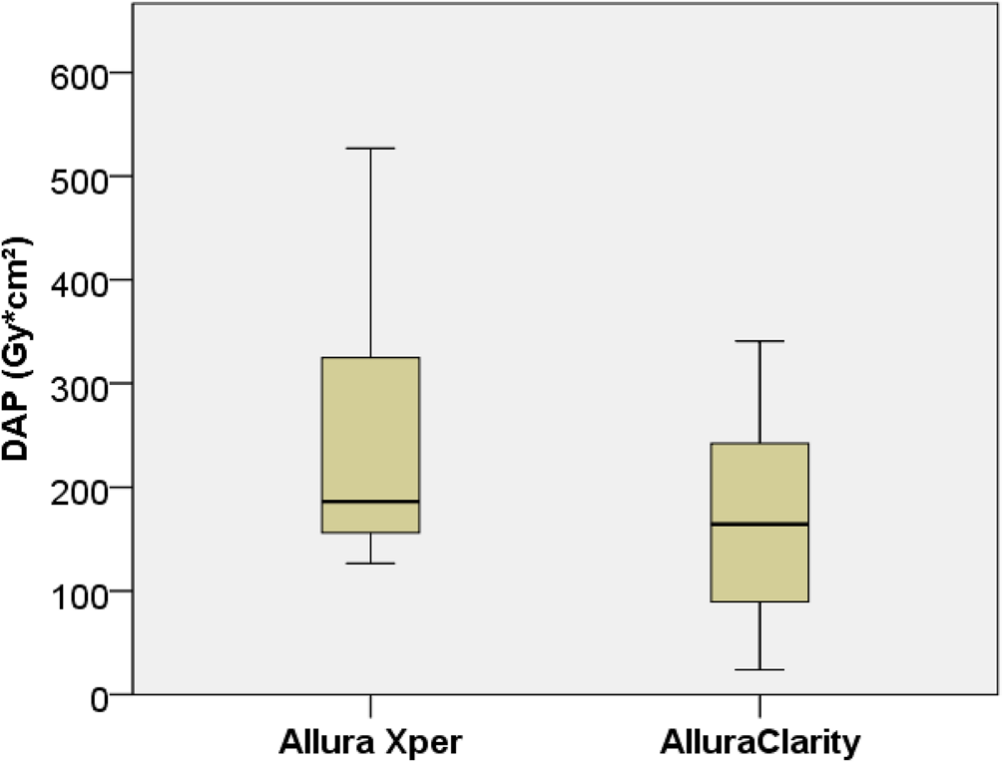
Box plot showing the mean cumulative dose area product (DAP) during adrenal vein sampling (AVS) for Allura Xper (235.1 ± 113.1 Gycm^2^) and AlluraClarity (170.1 ± 94.5 Gycm^2^). DAP includes both digital subtraction angiography and digital fluoroscopy.

After the technology upgrade from Allura Xper to AlluraClarity the mean DAP was reduced by 28% (235.1 ± 113.1 vs. 170.1 ± 94.5 Gy*cm^2^, p = 0.01) (Fig. 1). The mean AK also decreased by 35% (1.7 ± 0.7 vs. 1.1 ± 0.7 Gy, p = 0.01) after the technology upgrade from Allura Xper to AlluraClarity. There was no significant difference regarding the fluoroscopy time (36 ± 18 vs. 42 ± 23 min, p = 0.22) (Table 3).

### DSA image quality

**Figure 2:**
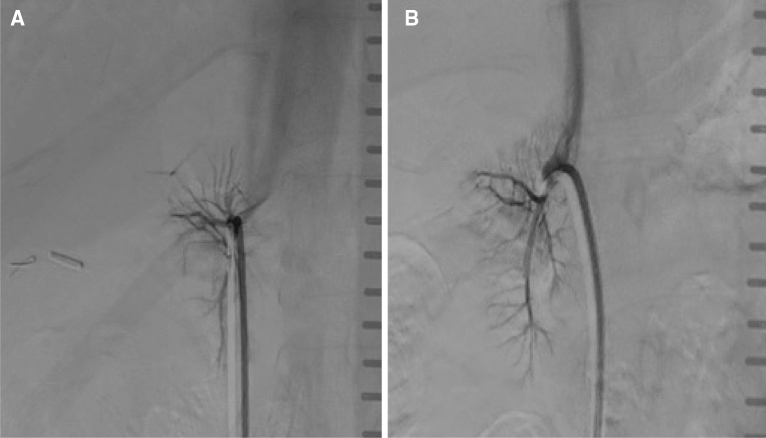
DSA of selectively catheterized right adrenal veins during AVS using Allura Xper (**A**) and AlluraClarity (**B**). A: 54y female, BMI 27.4 kg/m^2^ using Allura Xper with a cumulative dose area product of 205.2 Gycm^2^. B: 56y female, BMI 26.2 kg/m^2^ using AlluraClarity with a cumulative dose area product during of 169.7 Gycm^2^. Both observers rated the image quality for Allura Xper and for AlluraClarity as 4 = excellent including a sharp delineation of the adrenal veins with sufficient contrast, contrast stain of the adrenal gland and low level of subjective image noise.

Comparing Allura Xper and AlluraClarity no significant difference regarding DSA image quality could be observed with a median 3 vs. 3 (Interquartile range (IQR) 2–4 vs. 2–4, p = 0.67) in Fig. 2. Image quality ratings demonstrated a comparable level of inter-rater agreement for image quality ratings of AlluraClarity (k = 0.77) and Allura Xper (k = 0.75) (Table 4).

**Table 4:** DSA image quality of adrenal venograms.

Imaging technology	Average (IQR)	Reader 1 (IQR)	Reader 2 (IQR)	Weighted Kappa (95%-CI)
Allura Xper	3 (2–4)	3 (2–4)	3 (2–4)	0.75 (0.60–0.89)
AlluraClarity	3 (2–4)	3 (2–4)	3 (2–4)	0.77 (0.60–0.94)
p-value Allura Xper vs. AlluraClarity	0.67	0.94	0.52	

Data presented as mean with interquartile range (IQR) or with 95% confidence intervals (CI); * = significant. Image quality was graded as follows: 1, poor; 4, excellent.

## Discussion

Our study demonstrates that the AlluraClarity technology upgrade enables a radiation dose reduction up to one third in patients during AVS compared to the precursor technology Allura Xper without compromising image quality.

It is conceivable that the main reason for a significant reduction of radiation dose after the upgrade using AlluraClarity results from the additional filters of aluminum and copper during DSA and fluoroscopy. By changing system parameters AlluraClarity renders over 500 different acquisition settings with respect to the particular area of interest. Various studies have already demonstrated the potential in radiation dose reduction using AlluraClarity during intracranial angiography, cardiac angiography, iliac angiography or abdominal angiography^16–19^. Apparently, procedural settings during cerebral interventions focus more on the pixel shift feature during steady conditions, as thoracic and especially abdominal settings emphasis more on the motion control feature in order to reduce breathing artifacts^20^.

Previous studies of abdominal interventions such as transarterial chemoembolization (TACE) and transjugular intrahepatic portosystemic shunt (TIPS) using AlluraClarity abdominal settings reported dose reductions of > 50%^19, 21^. We observed a lower dose reduction of only 28% using the identical AlluraClarity abdominal settings during AVS.

The main reason for less dose reduction during AVS might be due the complexity of intervention. The most challenging part of AVS remains the reliable cannulation of the right adrenal vein (RAV)^10^. Identifying the RAV-ostium from the IVC with 1–2 mm diameters requires long fluoroscopy times. Previous studies revealed that the dose reduction provided by Clarity is lower in fluoroscopy mode (33–52%) than in DSA mode (73–79%)^17, 19^. Hence, the total dose reduction for a procedure depends on the proportion of each acquisition mode (DSA vs. fluoroscopy): A lower total dose decrease is expected for procedures with a high contribution of fluoroscopy to the total dose. Therefore, the relative long fluoroscopy time in our study (Allura Xper: 42 ± 23 min, AlluraClarity: 36 ± 18 min) explains a lower dose reduction in total DAP compared to previous dose reduction studies (Allura Xper: 7–26 min, AlluraClarity: 7–24 min)^17, 18^.

Furthermore, AlluraClarity provides three different levels of fluoroscopy-setups at the operator’s console ranging from low and medium to high dose settings. These changes can be applied differently for each fluoroscopy-run and might change due to individual preferences from operator to operator (Table 2). Unfortunately, these specific individual sub-settings are currently not monitored by the vendors dose reports and could not be tracked retrospectively. The default settings contain the low dose profile, which can be increased individually.

The relative increase in contrast agent used by 10% for additional DSA- or fluoroscopy runs may be caused by the verification of correct catheter placement before and after sampling (Table 3). Our results show a significant radiation dose saving potential after the upgrade to AlluraClarity with a mean DAP decreasing from 235.1 to 170.1 Gy*cm^2^. Recently the SPARTACUS multicenter trial released comparable mean AVS procedure doses from the identical fluoroscopy system AlluraClarity of 147 (1.1–1186) Gy*cm^2^^11^.

The main limitation of our study is the variety of different operators involved over 4 years due to its retrospective nature. Although AVS is to be considered as a highly standardized procedure, operators at different skill levels use their personal workflow of intervention during AVS. Evidently, identical and matched operators should have performed the interventions for both groups of patients before and after the technology upgrade.

Another limitation of our study is that we only investigated total DAP reduction. However, we were not able to separately analyze the reduction of DAP in fluoroscopy mode vs. reduction of DAP in DSA mode. Unfortunately, the separated DAP for DSA and fluoroscopy were not recorded during the study period and could not be retrospectively retrieved.

The image quality assessment is a further limitation of the study. Image quality and vessel contrast depend not only from the angiographic system, but also from the applied flow rate during injection and amount of injected contrast agent for each series. We believe that these incongruences regarding procedure workflow apply to both groups and do not result in a systemic bias. Although the image quality assessment had been adopted from well-established previous studies, it remains a subjective tool. However, the image-assessment results demonstrate a good inter-observer-agreement concerning AlluraClarity (k = 0.77) and Allura Xper (k = 0.75).

In summary, we have shown that the new angiographic imaging technology significantly decreases the radiation dose during adrenal vein sampling without compromising image quality or increasing fluoroscopy time or contrast volume.
